# A novel, scenario-based approach to comparing non-pharmaceutical intervention strategies across nations

**DOI:** 10.1098/rsif.2024.0301

**Published:** 2024-09-11

**Authors:** Justin M. Calabrese, Lennart Schüler, Xiaoming Fu, Erik Gawel, Heinrich Zozmann, Jan Bumberger, Martin Quaas, Gerome Wolf, Sabine Attinger

**Affiliations:** ^1^ Center for Advanced Systems Understanding (CASUS), Untermarkt 20, Görlitz 02826, Germany; ^2^ Helmholtz-Zentrum Dresden-Rossendorf (HZDR), Bautzner Landstraße 400, Dresden 01328, Germany; ^3^ Department of Ecological Modelling, UFZ—Helmholtz Centre for Environmental Research, Leipzig, Germany; ^4^ Department of Biology, University of Maryland, College Park, MD, USA; ^5^ Research Data Management—RDM, UFZ—Helmholtz Centre for Environmental Research, Leipzig, Germany; ^6^ Department Monitoring and Exploration Technologies, UFZ—Helmholtz Centre for Environmental Research, Leipzig, Germany; ^7^ Department of Economics, UFZ—Helmholtz Centre for Environmental Research, Leipzig, Germany; ^8^ Institute for Infrastructure and Resources Management, Leipzig University, Leipzig, Germany; ^9^ German Centre for Integrative Biodiversity Research (iDiv), Halle–Jena–Leipzig, Germany; ^10^ ifo Institute—Leibniz Institute for Economic Research, Munich, Germany; ^11^ Department of Computational Hydrosystems, UFZ—Helmholtz Centre for Environmental Research, Leipzig, Germany

**Keywords:** COVID-19, non-pharmaceutical intervention, modelling, epidemiological, behavioural, macroeconomic

## Abstract

Comparing COVID-19 response strategies across nations is a key step in preparing for future pandemics. Conventional comparisons, which rank individual non-pharmaceutical intervention (NPI) effects, are limited by: (i) a focus on epidemiological outcomes; (ii) NPIs typically being applied as packages of interventions; and (iii) different political, economic and social conditions among nations. Here, we develop a coupled epidemiological–behavioural–macroeconomic model that can transfer NPI effects from a reference nation to a focal nation. This approach quantifies epidemiological, behavioural and economic outcomes while accounting for both packaged NPIs and differing conditions among nations. As a first proof of concept, we take Germany as our focal nation during Spring 2020, and New Zealand and Switzerland as reference nations with contrasting NPI strategies. Our results suggest that, while New Zealand’s more aggressive strategy would have yielded modest epidemiological gains in Germany, it would have resulted in substantially higher economic costs while dramatically reducing social contacts. In contrast, Switzerland’s more lenient strategy would have prolonged the first wave in Germany, but would also have increased relative costs. More generally, these findings indicate that our approach can provide novel, multifaceted insights on the efficacy of pandemic response strategies, and therefore merits further exploration and development.

## 1. Introduction

The COVID-19 pandemic caught the world unprepared and exposed critical weaknesses in national pandemic response plans. Within four months of its emergence in Wuhan, China, the disease had spread globally, and the World Health Organization (WHO) upgraded COVID-19 to pandemic status in March 2020. Most national governments were not willing to allow the unchecked spread of SARS-CoV-2, but effective countermeasures were challenging and costly to implement. Non-pharmaceutical interventions (NPIs), such as mask mandates, lockdowns and school closures, quickly became essential tools for combating the incipient COVID-19 pandemic [1–4]. NPIs were, consequently, applied around the world, but in heterogeneous ways and with diverse outcomes. We therefore believe that retrospective comparisons among the contrasting response measures employed by different nations will identify the most effective intervention strategies and will facilitate better preparation for future pandemics.

Multiple studies have assessed NPI deployment across nations and have attempted to rank and evaluate NPIs in terms of their average epidemiological effectiveness [1,5–8]. Most commonly, NPIs are understood as effective if they impact specific epidemiological variables, in particular, if they reduce the basic reproduction number, $R_{0}$ , or COVID-19-related mortality [9–12]. Furthermore, several studies have compared how NPI effectiveness varied among nations [9,10], relying on datasets such as the Johns Hopkins Coronavirus Resource Center [13] or the Oxford COVID-19 Government Response Tracker [14]. Other studies have assessed NPI impacts in selected countries [15], on a regional scale [11,16], or examined effectiveness in individual countries [17,18]. Overall, NPIs such as school closures, restricting gatherings, banning public events or mandating masks were found to be effective by a range of studies [10,19].

While providing a useful baseline, such comparisons are difficult to perform and subject to a number of complications. First, NPI effectiveness has primarily been assessed from an epidemiological perspective, despite widespread evidence of marked variation in social and economic consequences of such interventions. A more balanced approach would also account for social and economic outcomes when accessing NPI performance (e.g. [15,20]; for sustainability implications refer to [21]). Second, NPI comparisons typically seek to isolate the effects of individual NPIs while assuming that the combined effects of multiple NPIs are additive, but there are very few situations in which individual measures have been applied in isolation. Instead, interventions tend to be applied as packages, where the package composition, the application sequence and the timing of application are all important and can introduce non-additive effects [22–25]. Finally, cross-national comparisons are frequently confounded by each country having its own set of circumstances including governance, financial and public health systems, as well as education levels, political attitudes, income distributions and myriad other factors, which we collectively refer to as national framework conditions (NFCs) [26].

Here, we propose a novel, scenario-based method of comparing NPI effectiveness across nations that treats NPIs as packages of interventions, can control for NFCs, and integrates economic and social considerations into the assessment. Our approach is based on a susceptible–infected–recovered (SIR) compartment disease model coupled to elemental behavioural [27] and economic models [28], and can be fit to data from different nations. Because data quantity, quality and availability vary markedly among nations, we aim to keep our models simple so that they can be reliably parametrized for as many nations as possible. Following Schüler *et al*. [29], we estimate the time-dependent disease transmission rate for each nation as a piece-wise constant function with breakpoints defined by the times of NPI implementation or removal. By treating bundles of interventions as a package impacting the overall time-dependent transmission rate, this approach eliminates the need to identify separate effects of individual interventions and shifts the focus to intervention strategies. It is then possible to quantify how a focal nation’s epidemiological, social and economic outcomes would have changed under the NPI strategy of a reference nation through ‘what-if’ scenario simulation. Specifically, we transfer a relativized version of the time-dependent transmission rate estimated from the reference nation into the focal nation’s model, simulate the epidemiological dynamics forward in time, and then calculate the resulting social and economic costs.

As a first proof of concept, we focus our case study on the initial COVID-19 wave in Spring 2020, well before vaccines became widely available. Additionally, we take Germany (DE) as our focal nation, and we consider scenarios from reference countries that employed more stringent (New Zealand, NZ) and less stringent (Switzerland, CH) NPI strategies relative to Germany. We find that the New Zealand scenario would have more quickly brought the first COVID wave in Germany under control, but at the expense of much larger reductions in social contacts than either the CH or DE scenarios. Additionally, compared with the strategy DE actually implemented, both the CH and NZ scenarios would have resulted in substantially higher economic costs, but for contrasting reasons. Finally, we demonstrate starkly different epidemiological outcomes based on differential social dynamics between countries, particularly when transferring the CH scenario to DE.

## 2. Materials and methods

### 2.1. Background: national framework conditions and spring 2020 COVID-19 response in Germany, New Zealand and Switzerland

Many NFCs are correlated with COVID-19 outcomes across nations. Specifically, the age structure, obesity, urbanization, GDP per capita, trust in the government, government effectiveness and health expenditures are NFCs with higher correlation coefficients [30–36]. It is therefore critical to control for variation in these important NFCs in cross-national comparisons, which we do here in two ways. First, we have chosen reference countries that are in many ways similar to Germany. Second, we transfer the relativized contact rates from the two reference countries to the focal country, which we describe in detail in §2.5.

**Figure 1.**
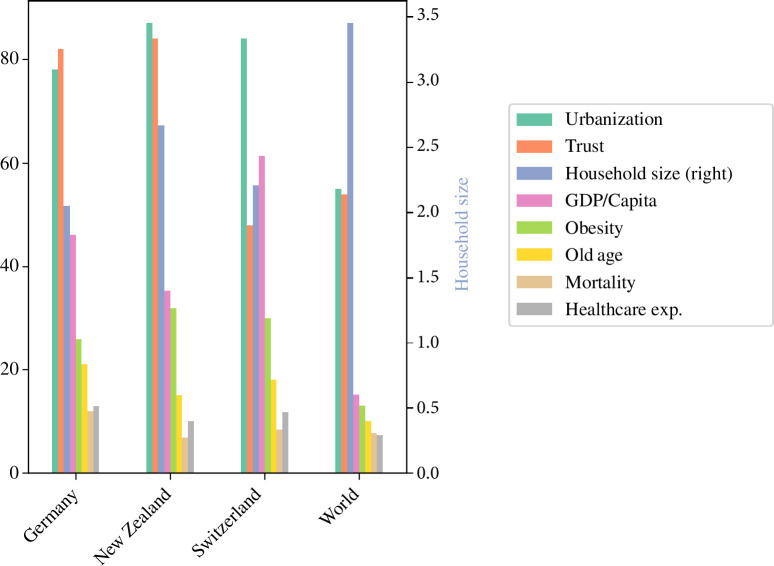
A comparison of some of the key NFCs between the reference country, the focal countries and the world. The NFCs are the percentage of the population living in an urban environment (Urbanization), the trust in the government (Trust), the average household size (Household size (right)), the GDP per capita per 1000 (GDP/capita), the prevalence of obesity (Obesity), the percentage of the population which is 65 years and older (Old Age), the mortality per 1000 per year (Mortality) and the healthcare expenditure in per cent of total GDP (Healthcare exp.).

Comparing key NFCs across our case study nations, we see that these three countries are quite similar in terms of age structure, obesity, urbanization and the trust in their governments compared with world averages (figure 1).

**Figure 2.**
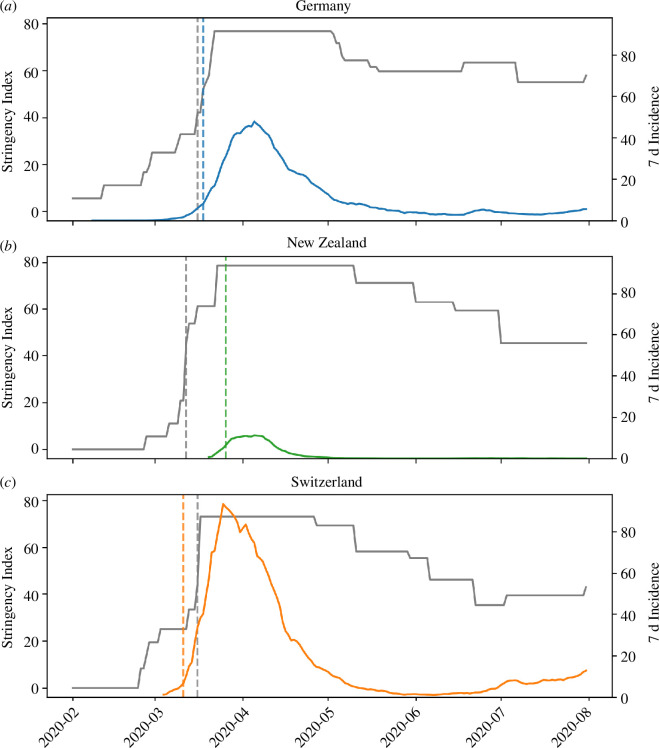
A comparison of the development over time of the stringency index (grey, left *y*-axis) and the 7 d incidence (right *y*-axis) for (*a*) Germany, (*b*) New Zealand and (*c*) Switzerland. The grey vertical lines indicate the day at which the stringency index surpasses the threshold of 40. The coloured vertical lines indicate the day at which the time derivative of the 7 d incidence surpasses the threshold of 1.5 d^−2^.

We identified key milestones and turning points in the national responses to the COVID-19 pandemic in early 2020 based primarily on the COVID-19 Government Response Tracker dataset [14], Our World in Data [37] and supplemented with sources specific to each nation [38–44]. Figure 2 shows, for each of the three nations, the 7-day incidence against the stringency index, which is a composite indicator tracking nine NPIs on a scale between 0 and 100 [14]. In electronic supplementary material, annex A, we break down the index into its nine constituents. A particular focus on comparing different response strategies was placed in the early days of the pandemic, as the reproduction number of the original variant of COVID-19 without intervention was estimated to exceed 1 in most cases [45,46]. Thus, not only the intensity of NPIs is relevant but the timing of their deployment is crucial.

Germany implemented NPIs in a step-wise manner (figure 2*a*), including the cancellation of large-scale events, on 10 March, the closing of schools and kindergartens in most federal states by 16 March, and culminating in a set of NPIs restricting private meetings and public life on 22 March [38]. During the same time, NZ (figure 2*b*) and CH (figure 2*c*) adopted a more stringent and a more lenient response, respectively. In CH, NPIs were deployed comparatively late, initially relying on public information campaigns and the restriction of large gatherings. Reacting to the strong increases in reported case numbers (figure 2*c*), CH implemented a variety of NPIs effective immediately on 16 and 17 March, including the closure of schools, non-essential workplaces and the banning of public events [47]. In contrast, NZ issued quarantine orders for international arrivals by 14 March, banned indoor gatherings of more than 100 individuals by 19 March, and enacted a detailed four-tiered pandemic plan by 21 March 2020 at comparatively low levels of infection. By 25 March, the highest alert level (‘Lockdown’) was announced, which banned all gatherings and travel, while mandating a strict stay-at-home order [43].

To account for differential onsets of the first COVID-19 wave, we now compare the timing of NPI implementation relative with the timing of fast growth in case counts across the three example nations. For each country, we therefore set a stringency index threshold of 40 and an incidence growth rate threshold of $1.5\;d^{-2}$ . Comparing the time lags between dates at which these thresholds were crossed, we see that NZ implemented strict NPIs 14 days before reaching the incidence growth threshold (figure 2*b*), while DE began NPI implementation only 2 days before crossing the growth threshold (figure 2*a*). In contrast, CH was slower to react and began NPI implementation in earnest 4 days *after* the growth threshold was exceeded (figure 2*c*). Though our chosen thresholds are somewhat arbitrary, the pattern of time lags across nations is robust to the choice of thresholds.

Following reductions in reported infection levels throughout the following weeks, all three nations responded with changes in NPI deployment. Germany and CH both gradually eased NPI stringency, albeit with differences in timing and the extent to which restrictions were lifted (cf. figure 2*a,c*): CH re-opened schools and non-essential commercial establishments earlier and permitted contacts and public events under fewer restrictions. In NZ, NPIs were adjusted based on the pandemic plan, reducing NPI stringency in three steps as the reported infection levels fell (figure 2*b*).

### 2.2. Coupled model analysis

Here, we define each component of our coupled epidemiological–behavioural–macroeconomic model, specify how the components interact, and then describe how we transfer estimated rate functions from reference countries to DE. We specifically aim to keep the complexity of our models at a minimum for multiple reasons. First, as our long-term goal is to compare many countries, we must be able to obtain and use the same kind of data for all countries we consider. This condition places hard limits on how much data are available for parametrizing our models. In other words, we cannot just focus on the handful of countries in the world with the most detailed data, but instead have set up our models to be usable and tractable based on the much sparser ‘lowest common denominator’ datasets that can be obtained across many nations. Second, we specifically aim to keep the complexity of our models at a minimum to preserve parameter identifiability, which allows us to directly link all model components to data. Finally, we can focus on the effects of the scenario transfers, which could be obscured by more complex models.

### 2.3. Epidemiological–behavioural model

We begin with a simple SIR compartment model to represent the epidemiological dynamics,


(2.1) $$\begin{array}{rl} \dot{S} & =-\frac{\beta_{j}}{N}IS \end{array},$$

(2.2) $$\begin{array}{rl} \dot{I} & =\frac{\beta_{j}}{N}IS-\gamma I \end{array},$$

(2.3) $$\begin{array}{rl} \dot{R} & =\gamma I \end{array},$$

where S , I and R are the number of susceptible, infected and recovered individuals, and N=S+I+R . The parameters of this model are the recovery rate γ and the piece-wise contact rate $\beta_{j}$ , where j indexes the dates at which national NPIs are introduced or lifted [29]. With this model, we assume post-infection immunity for the duration of the study, which is approximately five months. The post-infection immunity for the original SARS-CoV-2 variant is of the order of months [48]. And even if it would be shorter, at the end of the simulation period more than 99% of the population has not been infected in any of the countries or scenarios, making this a well-founded assumption.

To incorporate behaviour in our model, we note that an individual’s perceived risk of an infection, personal risk aversion, valuation of social contacts and potential for income loss may affect their decisions [49]. The resulting autonomous behavioural adjustments that individuals make in response to an outbreak, when averaged over the population, can substantially affect epidemiological dynamics [27]. Population-level differences in behavioural responses may therefore represent an important source of variation in COVID outcomes among nations. To capture this potential variation in our coupled model, we assume that individuals derive utility u from social contacts, and following [27], model utility as


(2.4) $$\begin{array}{ccc} & u(\beta_{r_{j}})=\frac{1}{1-\epsilon }(\tau_{j}^{1-\epsilon }\beta_{r_{j}}^{\epsilon }-\epsilon \beta_{r_{j}})\;, & \end{array}$$

with $\beta_{r_{j}}\in (0,1]$ being the relative contacts with respect to the pre-pandemic contact level at j th NPI period and ϵ∈(0,1) capturing how important it is to enjoy at least some contacts. The variables $\tau_{j}\in (0,1]$ model the contact reduction that results from the NPIs. If there are no NPIs, we set $\tau_{j}=1$ and the stricter the NPIs, the lower the value of $\tau_{j}$ . The level of social contacts that maximizes the utility is ${\bar{\beta }}_{r_{j}}=\tau_{j}$ , and the corresponding level of utility is normalized to $u({\bar{\beta }}_{r_{j}})=\tau_{j}$ . In an epidemic situation where almost all individuals are susceptible, the individual risk of an infection can be approximated by $\beta_{r_{j}}IS/N\approx \beta_{r_{j}}I$ .

A rational, risk-averse individual chooses their contacts as a trade-off between utility from contacts and the expected utility loss from an infection Δv (refer to electronic supplementary material, annex B). The number of contacts is determined by


(2.5) $$\begin{array}{ccc} & \max_{\beta_{r_{j}}}\left\{u(\beta_{r_{j}})-\beta_{r_{j}}I\Delta v\right\}\;. & \end{array}$$

Solving this yields


(2.6) $$\begin{array}{ccc} & \beta_{r_{j}}^{*}=\frac{\tau_{j}}{{\left(1+\frac{1-\epsilon }{\epsilon }\Delta vI\right)}^{\frac{1}{1-\epsilon }}}\;, & \end{array}$$

and with $\beta_{j}^{0}$ being the contact rates without the behavioural model, we can calculate the contact rates for the SIR model (2.1)–(2.3) with $\beta_{j}=\beta_{j}^{0}\beta_{r_{j}}^{*}$ . Following Quaas *et al.* [27], we set ϵ=0.7 . The parameters $\tau_{j}$ depend on the stringency of the j th NPI, whereas Δv depends on individual risk assessment and individual risk aversion and we assume it to be constant over the duration of our scenarios. This behavioural model does not describe NPI compliance. A visualization of equation (2.6) is provided in electronic supplementary material, annex B.

The NPI-related contact rates, $\tau_{j}$ , and the risk aversion, Δv , of the behavioural model are estimated inversely by solving a nonlinear least-squares problem for each nation individually. For all countries and scenarios, the recovery rate is set to the same, fixed value of $\gamma =0.14\;d^{-1}$ , which was obtained from the literature [50].

### 2.4. Macroeconomic model

The scenario analysis for economic activity is based on a model developed in Dorn *et al.* [28]. First, we model economic activity as a function of the time-dependent reproduction number, $R_{t}$ , following empirical relationships at the industry level to account for the heterogeneous effects of an aggregate NPI policy. Second, we simulate economic activity associated with different NPI scenarios. A key assumption in this analysis is that the reproduction rate $R_{t}$ is proportional to the aggregate NPI policy such that $R_{t}$ is lower the stricter the interventions.

To model economic activity as a function of $R_{t}$ , we measure economic activity over two time windows and link it to the associated reproduction numbers at those times. The first time window, $t_{1}$ , is immediately after the first lockdown began to be lifted. The second time window, $t_{2}$ , is after several step-wise relaxations of policy measures. We estimate the associated reproduction numbers as follows. First, we fit the SIR model (2.1)–(2.3) to the entire case data time series. Then, for each day in a given time window ( $t_{1}$ or $t_{2}$ ), we extract the model-fitted value of case numbers and estimate $R_{t}$ according to Cori *et al.* [51]. Finally, we average $R_{t}$ values over all days in a given time window. Repeating this procedure separately for $t_{1}$ and $t_{2}$ yields the estimates $R_{t1}$ and $R_{t2}$ . Finally, we regress measures of monthly economic activity $y_{m}(t_{1})$ and $y_{m}(t_{2})$ against $R_{t1}$ and $R_{t2}$ to estimate a linear relationship between economic activity and the reproduction number at the industry level.

Specifically, the German government announced partial re-openings on 20 April, so we set the first time window to be 27 April–2 May 2020 and estimated $R_{t1}=0.84$ (for comparison, the official Robert Koch Institute (RKI) nowcast value is $R_{t1,\mathrm{RKI}}=0.82$ [52]). Similarly, we estimated $R_{t2}=0.95$ ( $R_{t2,\mathrm{RKI}}=1.1$ ) over the second window from 9 to 14 June after several NPIs were lifted. Economic activity was measured at the industry level (NACE Rev. 2) based on the ifo Business Survey, a monthly survey among German firm managers that includes approximately 9000 responses with respect to their current and expected business activities (refer to [53] for details on survey methodology).

The second step in estimating the economic consequences of different NPI scenarios, which vary in their degree of stringency, is to simulate the economic activity associated with each scenario. All simulations start during the initial lockdown phase when $R_{t1}=0.84$ and then, for each set of contact rates considered, proceed forward in time until either the modelled number of newly reported cases falls below a threshold $I_{\max}$ or a maximum time threshold $T_{M}=300\;d$ is reached. The threshold $I_{\max}$ depends on the capacity of the public health system to control the epidemic through contact tracing and isolation. For Germany, this threshold was estimated early in the pandemic to be 300 newly reported cases per day, based on the number of health departments (refer to [28] for details). The length of time from shutdown initiation until one of the thresholds $I_{\max}$ or $T_{M}$ is reached, $T_{S}$ , defines the duration of lockdown, over which the economic costs are calculated. By calculating the duration of the lockdown this way, we implicitly make the assumption that $R_{t}<1$ on average after the relaxation of NPIs.

Each scenario is defined by time-dependent contact rates $\beta_{j}$ . For each set of $\beta_{j}$ , the behavioural–epidemiological model is simulated to obtain the lockdown duration and corresponding $R_{t}$ values throughout the lockdown. Given the $R_{t}$ values, the sector-specific economic activity can then be quantified throughout the lockdown through the regression estimated in step 1 above (refer to electronic supplementary material, annex C for full details). The total cost for scenario i is then obtained by summing over the lockdown period as


(2.7) $$C^{i}=\sum_{m=1}^{T_{end}}\sum_{k=1}^{16}100-y_{m}^{i,k},$$

where $y_{m}^{i,k}$ is the level of economic activity of sector k compared with pre-shutdown levels in scenario i and month m . Full details of how $y_{m}^{i,k}$ are calculated over the lockdown periods can be found in electronic supplementary material, annex C.

We then express the total cost for each scenario relative to a reference scenario in which $R_{t2}=R_{t1}$ and then plot total relative cost $C^{i}$ versus $R_{t2}^{i}$ . First, this allows us to visualize how the economic impact of a given NPI scenario, expressed as total relative cost, is driven by the stringency of lockdown measures, measured as $R_{t}$ . Second, this approach allows us to compare different NPI strategies based on where they fall along the relative cost curve.

### 2.5. Scenario transfer

To transfer the NPI strategy of a reference country to the focal country, we first identify the dates of NPI changes in both countries. Next, the model parameters are estimated for both countries separately through model fit to each country’s COVID-19 incidence data. For the scenario run, we use the first n=1 or n=2 NPI dates and associated disease transmission rates of the focal country, for the epidemic wave to partially evolve (i.e. the first n NPI dates of the focal country are fixed). The subsequent NPI dates and disease transmission rates are then transferred from the reference country to simulate how the wave would have further developed in the focal country under the reference country’s NPI strategy. To transfer contact rates between nations, we assume that NPIs have the same proportional effect in each nation, but NFCs cause the proportionality constants to differ between countries. We therefore calculate the relativized, transferred contact rates resulting from NPIs $\tau_{j}^{T}$ from the reference country (*R*) to the focal country (*F*) as


(2.8) $$\begin{array}{rl} \tau_{j}^{T} & =\tau_{j}^{F}\;\;\text{for }j\leq n \end{array},$$

(2.9) $$\begin{array}{rl} \tau_{j}^{T} & =\tau_{j-1}^{F}\frac{\tau_{j}^{R}}{\tau_{j-1}^{R}}=\tau_{j}^{R}\frac{\tau_{n}^{F}}{\tau_{n}^{R}}\;\;\text{for}\;j>n. \end{array}$$

The relativized contact rate transfers the effects of NPIs *per se*, but does not account for differences in autonomous behavioural responses between reference and focal nations. To account for behavioural differences, the estimated risk aversion parameter, Δv , can also be transferred from reference to the focal nation. To help separate these effects, we consider scenarios with only NPI transfer, and with both NPI and risk aversion transfer. We also note that our transfer method does not account for differences in compliance between nations.

To summarize, we take the initial conditions of the focal country $S_{0}^{F},I_{0}^{F}$ and the focal country’s transfer rates $\tau_{j\leq n}^{F}$ in order to set up the first wave as it occurred in the focal country. The actual scenario transfer takes place from the second or third NPI period onward (depending on n ) by taking the reference country’s transfer rates $\tau_{j>n}^{R}$ together with equation (2.9) to calculate the transferred $\tau_{j>n}^{T}$ . Depending on whether the risk aversion is also transferred, either $\Delta v^{F}$ or $\Delta v^{R}$ is used in the scenario run.

The model code is available from the accompanying code publication [54].

## 3. Results

**Figure 3.**
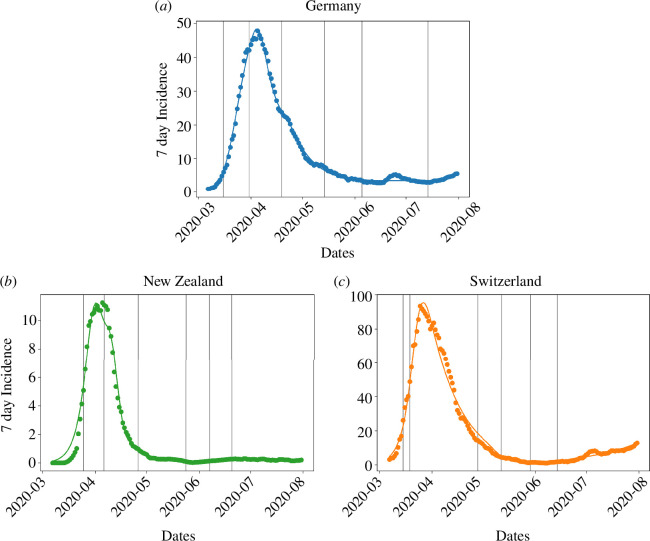
Fit of the coupled behavioural and epidemiological model to incidence data from (*a*) Germany, (*b*) New Zealand and (*c*) Switzerland during the first wave in Spring 2020.

The dynamics of the first COVID-19 wave in DE can be successfully reproduced with the coupled epidemiological (equations (2.1))–(2.3) and behavioural (equation (2.6)) model (figure 3*a*). Only the small summer peak could not be captured by the model, as it cannot be explained by NPIs. Similarly, the incidence curve for NZ can also be adequately reproduced (figure 3*b*), even though the initial increase in cases is so steep that the model cannot keep up and therefore creates a more gradual increase that, compared with the data, starts slightly earlier. The coupled model can describe the CH data as well, where there was a rapid initial increase in cases with a gradual decline thereafter (figure 3*c*). Overall, the epidemiological development during the first wave is qualitatively similar in DE, CH and NZ.

In all three cases, we were able to unambiguously identify reasonable estimates for all model parameters. We have verified that our parameter estimates were independent of the initial guesses that were used in our numerical search algorithm. In other words, the algorithm converged to the same estimates no matter what the starting values, suggesting that all model parameters are uniquely identifiable. Second, we also verified that *R* values calculated from the estimated model parameter are reasonable and are in general agreement with independent *R* estimates published by Germany’s public health agency RKI [52], which are based on a different methodology. Third, we show (see below) that the parametrized behavioural response estimated from our model agrees well with an independently estimated behavioural response from mobility data. Finally, our estimated model parameters agree well with published estimates of similar parameters from both Germany and other nations (table S2 in electronic supplementary material, annex D). Taken together, these checks suggest that the model parameters can be uniquely identified from the available data.

**Figure 4.**
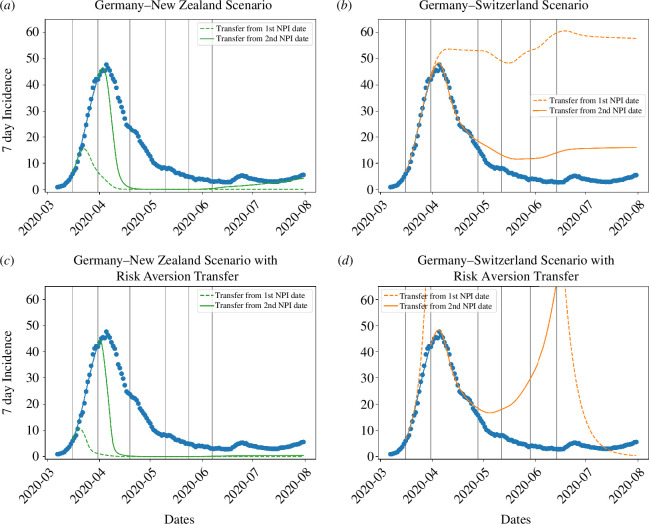
(*a*) New Zealand and (*b*) Switzerland scenarios transferred to Germany from the first (dashed line) and second NPI date (solid line) onward. The lower panels show the effect of also transferring the estimated risk aversion from the reference countries (*c*) New Zealand and (*d*) Switzerland to Germany.

Transferring the relativized transmission rate (equations (2.8) and (2.9)) from NZ to DE after the second NPI period onward (solid line) demonstrates that the first wave would have been stopped earlier than was actually observed in DE (figure 4*a,c*). Specifically, combining the NZ relativized transmission rate with DE’s level of risk aversion would have led to the outbreak ending in early May 2020 (figure 4*a*), while the more conservative level of risk aversion apparent in NZ would have helped end the outbreak by late April (figure 4*c*). Transferring the relativized transmission rate from the first NPI period onward (dashed line), the peak of the wave would have been significantly lower, at $15\;d^{-1}$ without risk aversion transfer and $11\;d^{-1}$ with risk aversion transfer. The outbreak would have also ended approximately one or two weeks earlier, depending on the risk aversion.

Applying the CH scenario to DE (figure 4*b,d*) after the second NPI period (solid line), we see that the first NPIs seem to be enough to break the wave. In the transfer scenario with DE risk aversion (figure 4*b*), the subsequent NPIs appear to be only stringent enough to create a dynamic balance with the 7 d incidence staying at approximately $15\;d^{-1}$ for approximately three months. Transferring after the first NPI period, the dynamic balance would have set in between incidence values of 50 and $60\;d^{-1}$ , which is slightly higher than the actual peak of the wave in DE. In stark contrast, the transfer scenario with CH risk aversion, which was markedly lower than for DE, would have resulted in a very large second wave during summer 2020 (figure 4*d*), no matter when the transfer starts. Comparing panels (*b*) and (*d*) in figure 4 shows that the autonomous behavioural response, which is governed by risk aversion, can qualitatively change the outcome of a given NPI regime when pronounced differences in risk aversion occur.

**Figure 5.**
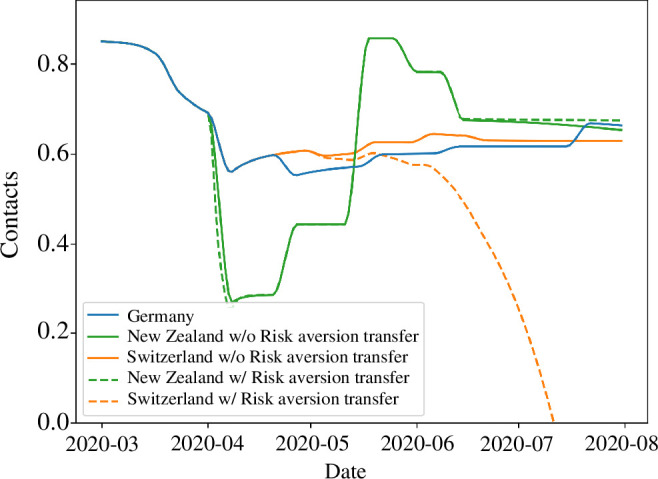
The dynamics of relative contact reduction for Germany and for the New Zealand and Switzerland transfer scenarios with and without risk aversion transfer.

The parametrized models also contain information on reductions in social contacts through the utility function (equation (2.5)). Specifically, the utility function quantifies the reduction in contacts relative to pre-pandemic levels. The actual DE NPIs and the DE risk aversion reduce contacts to a little less than 60% of pre-pandemic levels (figure 5, blue curve), and the CH scenario with DE risk aversion also causes a similar reduction in contacts (figure 5, orange solid curve). In contrast, the NZ scenario with DE risk aversion immediately reduces relative contacts to 30% from the onset of the first wave, which then gradually increases to values above the DE and CH scenarios, finally decreasing again to similar values as for DE and CH during summer 2020 (figure 5, green solid curve). Transferring the NZ risk aversion results in no important difference (figure 5, green dashed curve). The explosive increase in cases in the CH scenario with transferred risk aversion ultimately breaks the assumption of the behavioural model that the infection numbers are small, which results in relative contacts becoming negative during summer 2020 (figure 5, orange dashed curve). However, we have included this result (truncated at zero) both for the sake of completeness, and because it offers qualitative insight. Specifically, it seems reasonable that the large second wave would indeed drive contacts below the level observed during the first wave owing to the autonomous behavioural response. As an external check on our coupled model, we note that the relative contact reduction in DE estimated from our model (figure 5, blue curve) agrees remarkably well with the results presented by Rüdiger *et al.* [55, fig. 2*b*]. Importantly, they used mobile phone data to estimate a contact reduction of approximately 50%, whereas our estimates were based entirely on incidence data. The concordance between these differently estimated results suggests both that our behavioural sub-model is capturing the key drivers of contact reduction and is estimable from incidence data alone.

**Figure 6.**
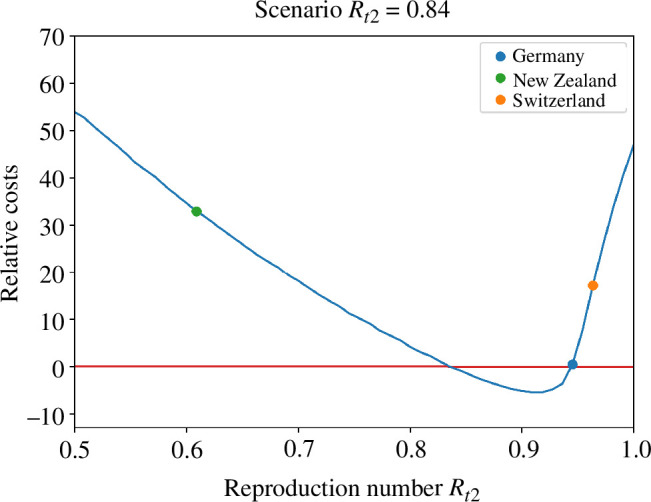
Relative economic costs of the three different NPI strategies in the context of Germany.

The relative cost function from our economic model is convex and asymmetric, showing that long-run costs increase faster and reach higher levels under very lenient NPI scenarios than under stricter NPI scenarios (figure 6). Economic activity is more sensitive to long shutdown duration (lenient NPIs) than to shorter but stricter shutdowns. Germany imposed measures that resulted in somewhat higher than optimal economic costs (figure 6, blue point). The minimum is found between the shutdown costs ( $R_{t1}=R_{t2}=0.84$ ) and partial opening costs ( $R_{t2}=0.95$ ), suggesting that economic costs would have been minimized if the partial opening measures in DE had been stricter compared with the actually implemented strategy.

Despite resulting in divergent epidemiological outcomes (figure 4), and similarly large differences in social outcomes (figure 5), the economic model suggests that both the NZ and CH scenarios, when viewed in the context of DE, were suboptimal and would have resulted in substantial relative cost increases (figure 6). Here, we focus only on the cases where German risk aversion was used in both the CH and NZ scenarios, as: (i) differences in risk aversion had negligible economic consequences in the NZ scenarios; and (ii) CH risk aversion resulted in $R_{t2}>1$ , which violates one of the economic model’s assumptions. In the case of NZ, the more stringent NPI response quickly brings the first wave to heel, but results in substantially increased short-term economic impacts (figure 6, orange point). In contrast, the more lenient CH strategy has lower initial economic impact, but the resulting prolonged higher case numbers cause costs to accumulate over the longer term, yielding even higher relative cost increases for the NZ scenario (figure 6, green point). All results, including the estimated parameters for all countries, are available in the accompanying data publication [56].

## 4. Discussion

Being better prepared for future disease outbreaks requires that we learn as much as possible from the COVID-19 pandemic. Cross-national comparative studies are foundational components of this learning process, but have been limited by a narrow focus on single-factor (typically epidemiological) outcomes. Such comparisons are further complicated by NPIs being applied as packages of interventions, which makes it more difficult to quantify the effects of individual measures in isolation particularly when interventions can interact nonlinearly. Finally, cross-national comparisons of intervention effects require special care as myriad differences among nations (i.e. NFCs) may contribute to broadly different outcomes. In contrast, we have taken a novel, scenario-based approach that explicitly considers the epidemiological, economic and social consequences of NPIs in the context of a focal country, recognizes the non-independence of NPIs as applied in practice, and can control for differences in NFCs. We emphasize that our current coupled model and results are intended as a first proof-of-concept of the viability of our approach. We thus proceed to interpret our results cautiously, and focus on highlighting what the current framework does well and what needs to be improved in subsequent efforts.

Our goals here were to develop an elemental, data-driven approach to scenario-based, inter-country comparisons of NPI response effects and to demonstrate the potential insights that can be gained from this framework. We have therefore kept our component models as simple as possible. As one example, our epidemiological model is based on the classical SIR model and therefore lacks additional compartments featured in some [57,58], but not all [29,59], COVID-19-focused compartmental models. As another example, our scenario transfer approach assumes equal compliance between focal and reference nations, as reliable data on compliance is not available in many countries. This focus on minimalism serves three pragmatic goals. First, it preserves parameter identifiability for all components of our coupled model, which is a prerequisite for performing empirically informed, cross-national comparisons. Indeed, while more intricate models might be easier to justify mechanistically, this additional detail will often come at the cost of parameter identifiability, which is an issue that frequently plagues complex compartment-type disease models [60,61]. Second, our focus on parsimony increases the number of countries that can be compared within our framework because it keeps the data requirements as low as possible. Given that data quantity, quality and accessibility vary tremendously across nations, it is extremely important to carefully balance data needs against model realism. Thus, any future developments of our framework must maintain a focus on the ‘lowest common denominator’ data that can be obtained for all countries of interest. Adding features and embellishments that cannot be supported by available data will only break the statistical tractability of the coupled model system and undermine its ability to yield reliable, data-driven insights. Third, by taking NPIs exclusively as bundled interventions and quantifying their aggregate effects on transmission rates, we circumvent the problem of having to describe and quantify the effect of each single measure. Furthermore, this approach aligns with the fact that most countries implemented bundles of NPIs simultaneously. Caution must, however, be taken to ensure that implementing and lifting these bundles in counter-factual scenarios happens in a logically consistent way, e.g. re-opening schools can only happen after they were closed.

Our approach focuses on controlled epidemics and we have employed approximations in the economic and behavioural sub-models that are well-behaved in the regions of phase space corresponding to this focus (i.e. where R<1 on average over the duration of the analysis). However, these approximations can break down and result in numerical problems when control efforts prove insufficient. For example, the economic model cannot handle scenarios that feature $R_{t2}>1$ , as happened in the CH scenario with CH risk aversion. This scenario also highlights a limitation of the behavioural model, which is the assumption of small case numbers I . This becomes problematic if we try to calculate the relative contact reduction from equation (2.5) (see figure 5, orange dashed curve). In the asymptotic case for I→∞ , not only the behavioural part, but also the epidemiological part of the coupled models (equations (2.1)–(2.3)) and (2.6) would break down. However, large I values are only gradually reached from June 2020 onward and the risk aversion parameter in equation (2.6) is generally small compared with the cases I , with $\Delta v<10^{-4}$ . This leads to a situation, where the equations remain stable until the point where the threshold $I>\frac{1}{\Delta v}$ is reached. The CH scenario with CH risk aversion exceeds this stability threshold from July onward. On the one hand, these numerical issues highlight areas for future model development. On the other hand, in cases such as that mentioned above for CH, we can immediately conclude that the focal control strategy has failed and thus do not need to consider it further.

A key step in taking our approach from its current proof-of-concept stage to use in planning and decision-making will involve properly quantifying parameter estimation uncertainty. In a parallel effort, Fu *et al.* [62] have developed a rigorous Bayesian estimation framework for complex dynamical systems with change-points where parameter values abruptly change at particular times. This statistical framework allows full uncertainty quantification, and was designed such that the change-points map directly onto NPI implementation dates which mark changes in disease transmission dynamics. However, embedding the coupled model and scenario transfer approach highlighted in this paper into the Bayesian framework established by Fu *et al*. [62] will require substantial further development of the coupled model, the statistical framework, and the code necessary to merge the two seamlessly. That remains work for the future, and is thus well beyond the scope of the present paper.

Choosing DE as our focal nation, and the first COVID-19 wave in Spring 2020 as our time horizon, we have considered two scenarios with contrasting properties. New Zealand implemented a very strict NPI strategy focused on intense national lockdown, while CH choose a much more lenient strategy specifically to avoid the economic and social consequences a hard nationwide lockdown entails. When transferred to the context of DE, these strategies produce markedly different epidemiological outcomes both relative to each other and relative to DE’s realized NPI strategy. We considered transferring response effects both after the first and after the second NPI period. The earlier a more (less) stringent response is transferred, the sooner it results in reduced (increased) infection numbers. However, the choice of transfer time did not fundamentally affect the results, and thus we focus below only on general patterns in the outcomes.

The NZ strategy results in a sharp, immediate decline in cases and near total elimination of the outbreak in a very short time. When the NZ NPI strategy is accompanied by the NZ level of risk aversion, which was higher than in DE, the outbreak is extinguished a couple of weeks sooner, but the qualitative pattern does not change. One important NFC we were unable to control for in these comparisons was the fact that NZ is an island nation. We thus urge caution in interpreting the NZ results, and suggest that both the relative cost and contact reduction estimates for the NZ transfer scenario represent limits in the sense that, for DE to have responded like NZ, it would have cost at least as much as the relative cost estimate, and would have reduced contacts at least as far as the contact reduction estimate. These limits follow from the fact that DE would have had to implement additional travel and entry restriction measures to offset the advantage NZ derives from being an island state. Implementing such measures would probably have resulted in both additional economic costs and further contact reductions.

Switzerland’s more lenient strategy produced contrasting results to the NZ transfer scenarios. Specifically, when coupled with DE risk aversion, the CH response stops further increases in cases, but also fails to drive incidence levels towards zero. Unlike the NZ scenarios, accounting for the lower level of risk aversion in CH compared with DE changes the outcome of the simulated transfer scenario both quantitatively and qualitatively. Specifically, a clear and much larger second wave appears following the first, indicating that the later-stage CH NPIs would have been inadequate to prevent a further outbreak in the context of DE. This qualitative shift occurs because the CH NPIs were at the limit of being able to contain a further outbreak (i.e. R≈1) in the DE context. Thus, a change in average individual risk aversion was sufficient to tip that fragile balance in the direction of another surge of infections (i.e. R>1 ).

The different strategies employed by DE, NZ and CH have substantially varied social costs. It is immediately apparent that the NZ strategy, when transferred to DE, would have resulted in dramatic reductions in social contacts, either with or without NZ risk aversion. Given the strong social and political reactions to lockdowns and loss of contacts that were actually observed in Germany, such further reductions could have been extremely disruptive. In the CH transfer cases, the difference in social costs, driven by differences between DE and CH levels of risk aversion, is striking. In the DE risk aversion case, the predicted reduction in contacts is comparable to what was actually observed in Germany, and much less than either of the NZ transfer scenarios, at least until mid-May 2020. However, in the CH transfer scenario with CH risk aversion, contacts are quickly driven to zero, resulting from a somewhat counter-intuitive feedback loop. Specifically, lower risk aversion interacts with weaker NPIs to produce much higher case counts. The exploding case numbers then drive an almost total loss of social contacts owing to individuals’ autonomous behavioural responses, even though risk aversion was markedly lower in CH than in DE. In other words, a severe enough outbreak can trigger dramatic reductions in social contacts through the autonomous behavioural response even when risk aversion is low (but non-zero).

When viewed through the lens of our DE-specific economic model, both the NZ and CH scenarios prove to substantially increase relative costs, but for contrasting reasons. Specifically, the NZ scenario incurs the immediate costs of a very strict lockdown, while only yielding modest epidemiological gains. In contrast, CH’s more relaxed approach gradually accumulates costs resulting from a sustained, low-intensity lockdown and concomitant reductions in business activity and efficiency. Relative to the ‘middle road’ strategy DE chose to implement, both of these reference approaches perform poorly from an economic perspective, while only NZ’s approach would have yielded epidemiological gains in the simulated DE national framework. Though we were not able to calculate relative economic costs in the CH scenario with CH risk aversion owing to a violation of the economic model’s assumptions, we can surely conclude the relative costs would have been even higher because it would have taken longer to bring the daily number of cases back down to the threshold $I_{\max}$ .

It is important to realize that the conclusions drawn from our scenario-based analyses are specific to a particular focal country, in this case, DE. Our approach is silent with respect to the optimality of the reference country’s strategy *in the context of the reference country*. For example, we tentatively concluded that DE’s realized NPI strategy achieved a more ideal balance between epidemiological and economic goals than NZ’s strategy would have, had it been implemented in DE. However, we draw no conclusions about how good NZ’s strategy was for NZ. Similarly, we also did not explore how well DE’s strategy would have performed in either NZ or CH. Doing so would require parametrizing our economic model for these countries, and then treating each as a focal country as we have done here for DE. Indeed, expanding the catalogue of both focal and reference nations to which our approach can be applied is a clear priority for subsequent work. We are therefore currently compiling the global databases that would support more extensive comparisons and intend to explore a broader range of case studies in the future.

We have developed a coupled modelling and scenario transfer framework and highlighted its utility with a proof-of-concept case study. As we have demonstrated, this framework can yield novel, empirically grounded and multifaceted insights into the efficacy of alternative disease control strategies. Furthermore, our approach facilitates equitable comparisons among strategies chosen by different nations. Finally, we have outlined clear lines along which our framework can be further developed into a general, robust platform for planning and comparing pandemic response strategies.

## Data Availability

Data files used in this article: [56]. Code used in our analyses: [54]. Supplementary material is available online [63].
